# Effect of Compactin on the Mycotoxin Production and Expression of Related Biosynthetic and Regulatory Genes in Toxigenic *Fusarium culmorum*

**DOI:** 10.3390/microorganisms10071347

**Published:** 2022-07-04

**Authors:** Alexander A. Stakheev, Denis V. Erokhin, Ekaterina A. Meleshchuk, Oleg D. Mikityuk, Natalia V. Statsyuk

**Affiliations:** 1 All-Russian Research Institute of Phytopathology, Bolshie Vyazemy, 143050 Moscow, Russia; erokhin.denis.v@gmail.com (D.V.E.); 7710069@mail.ru (E.A.M.); mod-39@list.ru (O.D.M.); 2 Shemyakin-Ovchinnikov Institute of Bioorganic Chemistry, Russian Academy of Sciences, 117997 Moscow, Russia

**Keywords:** zearalenone, deoxynivalenol, *Fusarium culmorum*, compactin, gene expression, mycotoxin production suppression

## Abstract

Zearalenone (ZEN) and deoxynivalenol (DON) are mycotoxins produced by various species of *Fusarium* fungi. They contaminate agricultural products and negatively influence human and animal health, thus representing a serious problem of the agricultural industry. Earlier we showed that compactin, a secondary metabolite of *Penicillium citrinum*, is able to completely suppress the aflatoxin B1 biosynthesis by *Aspergillus flavus.* Using the *F. culmorum* strain FC-19 able to produce DON and ZEN, we demonstrated that compactin also significantly suppressed both DON (99.3%) and ZEN (100%) biosynthesis. The possible mechanisms of this suppression were elucidated by qPCR-based analysis of expression levels of 48 biosynthetic and regulatory genes. Expression of eight of 13 *TRI* genes, including *TRI4*, *TRI5*, and *TRI101*, was completely suppressed. A significant down-regulation was revealed for the *TRI10*, *TRI9*, and *TRI14* genes. *TRI15* was the only up-regulated gene from the TRI cluster. In the case of the ZEN cluster, almost complete suppression was observed for *PKS4*, *PKS13*, and *ZEB1* genes, and the balance between two *ZEB2* isoforms was altered. Among regulatory genes, an increased expression of *GPA1* and *GPA2* genes encoding α- and β-subunits of a G-protein was shown, whereas eight genes were down-regulated. The obtained results suggest that the main pathway for a compactin-related inhibition of the DON and ZEN biosynthesis affects the transcription of genes involved in the G-protein-cAMP-PKA signaling pathway. The revealed gene expression data may provide a better understanding of genetic mechanisms underlying mycotoxin production and its regulation.

## 1. Introduction

*Fusarium* fungi are harmful pathogens infesting cereal crops in the temperate climatic zones and causing diseases such as Fusarium ear rot, Fusarium head blight, and various foot and root rots [1,2]. Some fungi of this genus may produce various mycotoxins, including deoxynivalenol (DON) and zearalenone (ZEN), which are among the most important and regulated mycotoxins at the global scale [3,4]. 

DON is considered to be the most common mycotoxin contaminating cereals worldwide [5,6,7]. The main mechanism of the trichothecene action is the inhibition of a protein biosynthesis in eukaryotic cells via binding to ribosomal peptidyltransferase [8,9]. DON and its derivatives induce apoptosis, affect intestinal and immune functions, cause reproductive disorders [10,11], and act as virulence factors in plants [12,13]. Another important fusariotoxin is zearalenone (ZEN), a xenoestrogen synthesized via a polyketide pathway [14]. ZEN is capable of binding to estrogen receptors in mammals, thus leading to reproductive disorders [15,16]. According to the International Agency for Research on Cancer, it is classified as a Group 3 carcinogen [17]. Several reports demonstrated that ZEN possesses neurotoxic, immunotoxic, and hepatotoxic properties [18,19,20]. 

Genes responsible for the DON and ZEN biosynthesis are organized in clusters. Each cluster consists of genes encoding enzymes, transcription factors, and transport proteins [21,22]. This type of organization significantly facilitates coordinated regulation of a cluster gene’s expression and its control. Genes involved in these clusters are under the control of specific regulators, in addition to global regulation factors mediating signals from different environmental stimuli.

In the case of the main DON producers, *F. graminearum* and *F. culmorum*, the core TRI cluster includes 12 genes, among which two (*TRI6* and *TRI10*) encode transcription factors and one (*TRI12*) encodes an efflux pump, involved in self-defense against the mycotoxin; the functions of *TRI9* and *TRI14* genes are still unknown [23]. The remaining seven genes encode enzymes responsible for different stages of a DON biosynthesis. In addition to the core cluster, there are at least two loci (*TRI1*-*TRI16* and *TRI101*) located in different parts of the genome, but also involved in the biosynthesis of this mycotoxin [24]. Some researchers considered the *TRI15* gene, which encodes a regulatory Cys2His2 zinc finger and is located outside the core cluster, to be another locus related to trichothecene production [25]. Genes crucial for ZEN biosynthesis include *PKS4*, *PKS13*, *ZEB1*, and *ZEB2*, which encode reducing and non-reducing polyketide synthases, isoamyl alcohol oxidase, and transcription factor, respectively. These neighboring genes form a cluster, coordinately regulated at the transcriptional level [26,27,28]. 

Traditional management of fungal diseases and mycotoxin contamination of food and feed is based on complex approaches, which include crop rotation, use of resistant cultivars, and application of fungicides. At the same time, the first two approaches are unable to completely prevent mycotoxin accumulation, while fungicidal treatments, especially in inadequate doses, may result in a chemical stress in attacked fungi, causing even more active synthesis of mycotoxins [29]. In addition, fungicides may have negative effects on human and animal health, and may lead to the emergence of resistant pathogen lines. From this point of view, natural compounds of microbial or plant origin able to inhibit mycotoxin production without suppression of fungal growth represent a promising alternative to synthetic fungicides, primarily in terms of safety and prevention of the development of resistance [30,31,32]. 

Earlier we showed that 6-demethylmevinolin (6-DMM or compactin), produced by *Penicillium citrinum* and known as an inhibitor of HMG-CoA reductase regulating cholesterol biosynthesis, was able to efficiently block the biosynthesis of a polyketide mycotoxin aflatoxin B1 (AFB1), and prevent melanin production and spore formation in *Aspergillus flavus* [33]. Interestingly, no other known inhibitor of mycotoxin biosynthesis provides such a triple effect. Since both AFB1 and melanin are synthesized in *A. flavus* via the polyketide pathway, we suggested that the revealed effect of compactin may be determined by its interaction with enzymes or regulating proteins of this biosynthetic pathway and, therefore, may be also applied to other polyketide mycotoxins. A preliminary study confirmed the inhibiting activity of compactin towards ZEN biosynthesis [34]. The purpose of this study was elucidation of the mechanisms of this phenomenon via the analysis of the effect of compactin on the levels of expression of various genes involved in ZEN production. Since the toxigenic strain of *F. culmorum* used in this study was also able to produce DON, a possible compactin effect on the DON production and expression of genes from the corresponding biosynthetic and regulatory clusters was additionally investigated. The results of the study demonstrated that the inhibitor acts at the transcriptional level, down- and up-regulating biosynthetic and regulatory genes involved in both ZEN and DON production. The obtained data are important for the development of new plant protection strategies, and for the better understanding of genetic mechanisms underlying mycotoxin production and its regulation. 

## 2. Materials and Methods

### 2.1. Fungal Strains and Culture Conditions

A toxigenic *Fusarium culmorum* strain FC-19 was provided by the State Collection of Plant Pathogenic Microorganisms of the All-Russian Research Institute of Phytopathology (Bolshie Vyazemy, Russia). A stock culture of the pathogen maintained on potato dextrose agar (PDA) slants was resumed by culturing for two weeks at 25 °C in Petri plates with the same medium to obtain actively growing and spore-producing colonies. Conidia were collected from a colony surface by flooding the mycelium with sterilized water and gently rubbing with a glass rod. The obtained spore suspension was filtered through sterile cotton wool to remove mycelium debris and adjusted to 1 × 10^5^–1 × 10^6^ conidia/mL to be used as the inoculum.

Submerged cultures of FC-19 used in the experiments were grown in 250 mL flasks with 50 mL of a liquid Myro medium of the following composition (g/L): soybean flour, 2.5; sucrose, 40; glycerol, 10; (NH_4_)_2_HPO_4_, 1; KH_2_PO_4_, 3; MgSO_4_, 2; NaCl, 5 (pH 5.9). Compactin (Sigma-Aldrich, St.-Louis, MO, USA) diluted in a minimal volume of ethanol was added into the autoclaved medium prior to inoculation up to a final concentration of 25 μg/mL, which, according to the preliminary experiments, had no inhibitory effect on the fungus growth, but completely suppressed ZEN production; for DON variants, the tested compactin concentrations were 10, 20, and 50 μg/mL. The equivalent amount of ethanol was added as the control. 

After inoculation with 2 mL of conidial suspension, flasks were incubated on an Infors HT RT-TK rotary shaker (Informs AG, Bottmingen, Switzerland) at 25 °C and 250 rpm for 4 (gene expression analysis) or 8 (mycotoxin analysis) days. All experimental and control variants were arranged in three replications.

### 2.2. Fungal Biomass and Mycotoxin Production Determination

To confirm the inhibitory effect of compactin, the content of ZEN and DON in control and compactin-treated FC-19 cultures was determined by HPLC using a Waters 1525 Breeze HPLC system equipped with a WAT 045905 detector (Waters Corp, Milford, MA, USA). At the end of the 8-day cultivation, each flask with a fungal culture was supplemented with 50 mL of dichloromethane and incubated for 1 h on a shaker under the same conditions. Then, the mycelium was separated by filtration, dried to a constant weight at 60 °C, and weighed to determine its dry weight. The filtered extract was passed through a layer of anhydrous sodium sulfate and evaporated at 40 °C on a Rotavapor R110 rotary evaporator (BÜCHI Labortechnik AG, Essen, Germany). The obtained residue was dissolved in a minimum volume of the mobile phase (see below), and a 10 μL aliquot of the prepared solution was applied to a temperature-controlled (27 °C) Symmetry C18 column (5 µm; 4.6 × 150 mm; Waters Corp., Milford, MA, USA). ZEN and DON elution from the column was performed using an acetonitrile:methanol:water mixture at the ratio of 1:1:0.75 (ZEN) or 1:1:4 (DON) as the mobile phase and a flow rate of 0.8 mL/min. The detection was performed at 254 nm. The mycotoxin content was quantified using commercial ZEN and DON preparations (Sigma-Aldrich, St.-Louis, MO, USA) as the standards. 

### 2.3. RNA Isolation and Reverse Transcriptase-Polymerase Chain Reaction

A total RNA was isolated from 4-day FC-19 cultures grown in the presence or absence of compactin (10 or 25 μg/mL for DON and ZEN gene clusters, respectively) using an RNeasy^®^ Plant MiniKit (QIAGEN, Hilden, Germany) in accordance with the manufacturer’s recommendations. A preliminary homogenization of the mycelium was carried out using liquid nitrogen. During isolation, samples were treated with an RNase-free DNAse set (QIAGEN, Hilden, Germany) according to the manufacturer’s instruction. The quality of the isolated RNA was evaluated spectrophotometrically using a Qubit 4 fluorometer (Thermo Fischer Scientific (Waltham, MA, USA) and by electrophoresis in 1% agarose gel. The reverse transcription reaction was performed using a QuantiTect^®^ Reverse Transcription Kit (QIAGEN, Hilden, Germany). The possible presence of DNA impurities was additionally assessed via amplification of cDNA using a pair of primers complementary to two exons of the *TEF1α* gene followed by electrophoresis. The presence of genomic DNA was determined by the size of the specific product (~600 or ~250 bp in the presence or absence of introns, respectively). Samples suspected to contain DNA contamination were not used in the further study.

### 2.4. Gene Expression Analysis by qPCR

The design of primer sets and the corresponding hydrolysis (TaqMan^®^ [35]) probes were conducted based on the genomic data of *Fusarium graminearum* strain PH-1 (GenBank accession numbers CP087871-74) and the search for potential homologues of the corresponding genes in the genome of *Fusarium culmorum* strain Class2-1B (GenBank accession numbers CP064747-50). Primers and probes were universal for both of these species. Oligonucleotides for the detection of transcripts of *PKS4*, *PKS13*, *TRI5,* and *TRI6* genes were described earlier [36]. Primer and probe sequences are listed in Table S1.

The qPCR reactions were carried out using a DT-96 thermocycler (DNA-Technology, Moscow, Russia) and the following universal amplification conditions: 94 °C for 1 min followed by 45 cycles at 94 °C for 10 s, 64 °C for 30 s, 72 °C for 5 s, and, finally, 72 °C for 5 min. The reaction mix was described in [37]. The total volume of a cDNA sample added to the reaction mix was 2 µL.

Two independent experiments were carried out, and each included three biological replicates.

### 2.5. qPCR Data Analysis

Amplification efficiency was calculated using the Cq slope method (see Table S1). The relative expression of target genes was estimated according to the mathematical model proposed by Pfaffl [38] using a QGene software package [39]. Results are represented as fold changes in treated samples calculated with respect to the same gene in the control sample (considered as 1.0).

### 2.6. Statistical Data Treatment

The statistical analysis of the obtained data was carried out using the Statistica 6.0 software package (StatSoft Inc., Tulsa, OK, USA). The significance of differences (*p* < 0.05) in the means between the experimental and control values were determined using a *t*-test for independent variables.

## 3. Results

### 3.1. Effect of Compactin on the ZEN and DON Production by F. culmorum FC-19

The results of the evaluation of the compactin effect on the ZEN and DON production by FC-19 are shown in Table 1. Like in the preliminary study [34], compactin demonstrated a significant inhibiting activity towards ZEN production. Addition of 25 μg/mL of compactin completely suppressed ZEN production by FC-19, since the residual ZEN content was below the limit of detection (2.0 ng/mL); at the same time, its negative effect on the mycelium growth was rather insignificant (~8% of the control).

No DON production was detected in the presence of 20 and 50 μg/mL of compactin. A very low DON content (<1% of the control) was observed for the lowest studied compactin concentration (10 μg/mL), while no significant effect on the fungal growth was revealed. Based on these results, this concentration (10 μg/mL) was used in the further gene expression experiments.

### 3.2. Effect of Compactin on the Expression of Genes Responsible for the ZEN Biosynthesis and Regulation in F. culmorum FC-19

**Figure 1:**
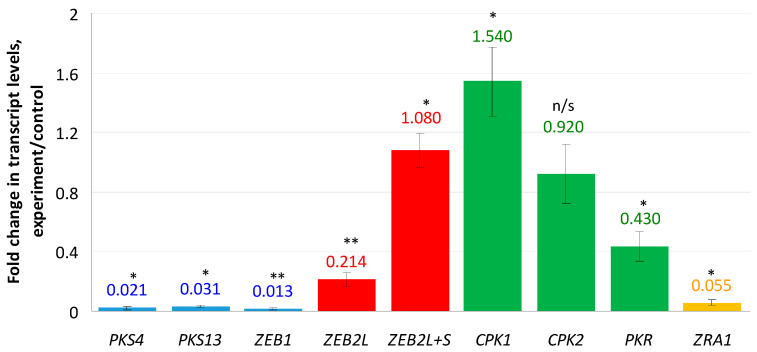
Effect of compactin on the relative expression of genes responsible for ZEN biosynthesis and regulation. The expression level (EL) for the untreated control was considered as 1.0; EL above or below 1.0 means up- or down-regulation, respectively. Blue bars indicate genes encoding biosynthetic enzymes; red bars indicate transcripts of a regulatory *ZEB2* gene; yellow bar indicates a *ZRA1* gene encoding the transport protein; green bars indicate genes encoding cAMP-dependent protein kinase subunits. Each bar is shown as M ± SD; n/s, no significant changes; * *p* < 0.05; ** *p* < 0.01.

Genes encoding enzymes responsible for the ZEN biosynthesis were shown to be almost completely down-regulated (Figure 1): 0.02 fold change (f.c.) for *PKS4*, 0.03 f.c. for *PKS13,* and 0.013 f.c. for *ZEB1*. The most interesting results were obtained for the transcription factor ZEB2 encoded by the *ZEB2* gene and regulating expression of ZEN biosynthetic genes. This factor is represented by two isoforms: ZEB2L (long) and ZEB2S (short). Since it was impossible to design a pair of primers specific to the *ZEB2S* isoform, we used two primer pairs: the first pair was specific for the “long” isoform, and the second pair was able to detect both “long” and “short” isoforms. As a result, we found a 0.214-fold decrease in the ZEB2L isoform expression. In contrast, when tested with the second primer pair, the expression level was increased (1.08 f.c.). This result can be explained by enhanced transcription of the *ZEB2S* isoform and suppressed transcription of the *ZEB2L* isoform under the compactin treatment conditions. In addition, expression of a *CPK1* gene encoding the catalytic subunit 1 of cAMP-dependent protein kinase (PKA), which regulates ZEN production, was increased 1.54-fold, whereas the change in the expression of the *CPK2* gene was insignificant (0.92 f.c., *p* > 0.05). The *PKR* and *ZRA1* genes encoding the PKA regulatory subunit and the ABC transporter required for ZEN production, respectively, were down-regulated (0.43- and 0.05-fold, respectively).

### 3.3. Effect of Compactin on the Expression of Genes Responsible for the DON Biosynthesis and Regulation in F. culmorum FC-19

**Figure 2:**
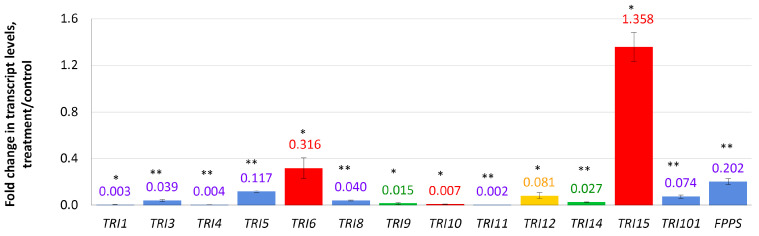
Effect of compactin on the relative expression of genes responsible for DON biosynthesis and regulation. The expression level for the untreated control was considered as 1.0. EL above or below 1.0 means up- or down-regulation, respectively. Blue bars indicate genes encoding biosynthetic enzymes; red bars indicate genes encoding regulation factors; yellow bar indicates the *TRI12* gene encoding transport protein (TRI efflux pump); green bars indicate genes with unknown functions. Each bar is shown as M ± SD; * *p* < 0.05; ** *p* < 0.01.

In this experiment, relative expression levels of 13 *TRI* genes related to the DON biosynthesis, and a *FPPS* gene encoding farnesyl pyrophosphate synthase, were analyzed. Four days after the addition of compactin to the cultivation medium, the expression of eight *TRI* genes was almost completely suppressed (Figure 2). The most inhibited gene was *TRI11* (0.002 f.c.) followed by *TRI1*, *TRI4* (0.003 f.c. each), and *TRI10* (0.007 f.c.) genes. The *TRI5* gene encoding trichodiene synthase (the first enzyme of the trichothecene biosynthetic pathway) was 0.12-fold down-regulated. A global regulator-encoding *TRI6* gene was the least down-regulated gene of the *TRI* cluster (0.316 f.c.). Two genes with unknown functions (*TRI9* and *TRI14*) were down-regulated (0.014- and 0.026-fold, respectively). The only up-regulated gene among those analyzed was *TRI15* (1.36 f.c.).

### 3.4. Effect of Compaction on the Expression of Key Regulatory Genes in F. culmorum FC-19

**Figure 3:**
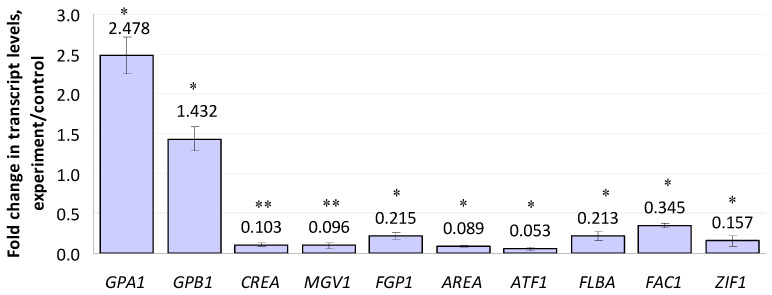
Effect of compactin on the relative expression of genes encoding regulatory factors. The expression level for the untreated control was considered as 1.0. EL above or below 1.0 means up- or down-regulation, respectively. Each bar is shown as M ± SD; * *p* < 0.05; ** *p* < 0.01.

Ten of the regulatory genes included in the study were up- or down-regulated by compactin (Figure 3). Among these, the expression of genes encoding α- and β-subunits (*GPA1* and *GPB1*) of a heteromeric G-protein showed a 2.47- and 1.43-fold increase, respectively. Note that the changes in the expression levels of genes encoding other Gα subunits (*GPA2* and *GPA3*) were insignificant (Table S2). The most down-regulated regulatory gene was *ATF1* (0.053 f.c.), which encoded a transcription factor; it was followed by *AREA* (0.089 f.c.), *MGV1* (0.096 f.c.), and *CREA* (0.103 f.c.). The *ZIF1* gene encoding a b-ZIP transcription factor demonstrated a 0.16-fold down-regulation. The expression levels of *FLBA* (encoding a regulator of G-protein signaling) and *P1* (encoding a Wor1-like protein) genes showed a 0.21- and 0.22-fold decrease, respectively. The least-inhibited gene (0.345 f.c.) was *FAC1* encoding adenylate cyclase. Fifteen other regulatory genes, including those of the velvet regulatory complex (*VE1*, *VELB*, *LAEA*), were insignificantly affected by compactin (Table S2).

## 4. Discussion

An appropriate mycotoxin control in food and feed products is a very important problem in modern agriculture. From this point of view, prevention of its production and accumulation represents a good practical approach. Since the existing pre-harvest technologies for the control of toxigenic fungi are unable to completely prevent mycotoxin contamination of agricultural products, the development of efficient and safe post-harvest approaches, including suppression of mycotoxin production or biodegradation of already accumulated mycotoxins, is of great interest. Furthermore, the majority of studies in this area are focused on biodegradation aspects, whereas the search for potential efficient inhibitors of mycotoxin production among natural compounds of plant or microbial origin seems to be underestimated. To date, the number of such studies is relatively low, and these are mainly focused on the AFB1 biosynthesis inhibitors (see, for example, [40,41]). Moreover, some compounds reported to suppress mycotoxin biosynthesis also possess antifungal activity; thus, it often remains unclear if they reduce mycotoxin content via suppression of its biosynthesis, or simply by reducing the mycelial biomass producing them.

Biosynthesis of mycotoxins and its regulation is a complex process involved in global regulatory networks and controlled by different factors [42]. To elucidate the possible mechanism of inhibition of DON and ZEN biosynthesis by compactin, a qPCR-based system for the estimation of relative expression of 48 genes was developed. The analyzed genes included those located in TRI and ZEN biosynthetic clusters, and genes encoding global regulators. Surprisingly, our study showed compactin to be able to suppress production of DON belonging to another group of mycotoxins, whose biosynthetic pathways differ from those of polyketide mycotoxins (AFB1 and ZEN). The obtained results demonstrated that the expression of eight of 13 TRI genes (*TRI7* and *TRI13* genes are non-functional in DON-producing strains [43] and, therefore, were excluded from the analysis) was almost completely repressed. The most inhibited genes included those responsible for the early stages of the trichothecene biosynthesis (*TRI4, TRI5, TRI101*). These data correlate well with the data of the earlier studies demonstrating the down-regulation of these genes in *F. graminearum* [44] and *F. culmorum* [45] by phenolic acids possessing inhibiting activity towards mycotoxin biosynthesis. However, the authors of the mentioned studies analyzed only eight and seven genes, respectively. In our study, we also examined other genes involved in DON biosynthesis. Among the genes encoding specific regulators of the TRI biosynthetic pathway, *TRI10* was significantly more suppressed compared to *TRI6*. This fact probably indicates that *TRI10* or one of its regulators represents the main target of a compactin-related inhibition mechanism. This suggestion is supported by a report on the strong down-regulation of genes coordinated by *TRI10*, such as *TRI3, TRI11*, and *TRI12* [46]. Another interesting result is the almost complete inhibition of transcription of *TRI9* and *TRI14* genes, whose functions are unknown. Dyer et al. 2005 [47] demonstrated that *TRI14* is required for DON biosynthesis and pathogen virulence in planta, but not in vitro. At the same time, expression of *TRI9* and especially *TRI14* in *F. graminearum* was significantly increased by agmatine, a compound promoting DON biosynthesis [48]. These genes probably encode unique *Fusarium*-specific regulation factors, whose exact functions are yet to be elucidated. Among the studied genes, *TRI15* was the only upregulated gene from the TRI cluster. This gene is predicted to encode a Cys2His2 zinc finger protein, but its role in a trichothecene biosynthesis is also unclear. Disruption of *TRI15* in *F. sporotrichioides* did not affect the synthesis of a T-2 toxin [25], but Ponts et al. (2007) suggested it can negatively regulate DON biosynthesis in *F. graminearum* [49]. The up-regulation of the *TRI10*-controlled *TRI15* gene can be explained by the presence of an unknown alternative regulation mechanism.

A qPCR-based approach helped to decipher a possible transcriptional mechanism of a compactin-related inhibition of a ZEN biosynthesis. In the case of *F. graminearum*, expression of *PKS4*, *PKS13*, and *ZEB1* genes is controlled by a ZEB2 transcription factor [50]. ZEB2 can be translated in two isoforms, ZEB2L (long) and ZEB2S (short). MRNAs encoding these isoforms are transcribed via an alternative promoter. Earlier it was shown that ZEB2L acts as an activator of genes from the ZEN biosynthetic cluster, whereas ZEB2S inhibits them. Genes responsible for the ZEN biosynthesis are transcribed only in the presence of a ZEB2L isoform alone. Under ZEN-inducing conditions, *ZEB2L* transcript was detected on the 3rd day of incubation, reaching the peak value by the 6th day. *ZEB2S* transcript appeared after 5 days of incubation and was present up to the 10th day. In our study, *ZEB2L* expression measured on the 4th day of the FC-19 incubation in the presence of compactin was significantly decreased (0.214 f.c.). At the same time, a transcript detected using primers specific for both isoforms demonstrated a slight up-regulation (1.08 f.c.). These data indicate that, in the presence of compactin, the ratio between L and S isoforms changes, and the S isoform inhibits ZEN biosynthesis via suppression of transcription of *PKS4*, *PKS13*, and *ZEB1* genes. Another ZEB2-controlled down-regulated gene was *ZRA1*, which encodes the ABC transporter that is also important for the ZEN biosynthesis [51]. In *F. graminearum*, ZEN production is controlled via the G-protein-cAMP-PKA signaling pathway [52]. In our study, the *CPK1* gene, encoding the subunit 1 of cAMP-dependent protein kinase, was up-regulated, whereas expression of the *CPK2* gene was not significantly changed, and a regulatory subunit-encoding *PKR* gene was down-regulated. These results are in accordance with the data obtained by Park et al. (2016), who demonstrate that CPK1 and PKR are negative and positive regulators of the ZEN biosynthesis, respectively [53].

An increased expression of α- and β-subunits of a heterotrimeric G-protein reaffirms the principal role of a compactin-related effect of G-protein-cAMP-PKA pathway on the inhibition of a mycotoxin biosynthesis in *F. culmorum*. According to Yu et al. (2008), *GzGPA1* and *GzGPB1* genes negatively regulate mycotoxin production in *F. graminearum* [54]. Genes encoding some regulators of a G-protein signaling pathway, such as RGS protein (*FLBA*) or adenylate cyclase (*FAC1*), were also shown to be down-regulated. At the same time, expression of the *RAS1* gene, which encodes Ras GTPase and is suggested to play an important role in the cAMP signaling pathway [55], was not significantly changed in the compactin-treated sample. Among other results of our study, attention should be paid to a significant down-regulation of the *AREA* and *CREA* genes encoding transcription factors related to the metabolism of nutrients [21,42]. This fact indicates that the mechanism of compactin-related inhibition of the mycotoxin biosynthesis in *F. culmorum* is more complex and involves various factors and metabolic networks. Interestingly, genes encoding proteins of an important velvet regulatory complex (*VE1, LAEA, VELB*) were not affected by the compactin treatment.

To date, the landscape of studies describing efficient inhibitors of ZEN and DON is relatively sparse. Reported compounds possessing such activity include some phenolic and flavonoid compounds of plant origin, antibiotics, and other microbial metabolites (see, for example [31,56,57,58,59]). The vast majority of such studies was carried out under in vitro conditions, although some phenolic compounds were also tested on wheat spikes [32]. As far as the authors are aware, there are no reports revealing any compounds able to efficiently inhibit different types of mycotoxins. From this point of view, the results obtained in our study are novel and of great interest. The revealed property of compactin, namely, the complete suppression of the biosynthesis of polyketide (AFB1 [33] and ZEN) and trichothecene (DON) mycotoxins at concentrations not influencing fungal growth, was confirmed for both in vitro conditions (compactin-containing nutrient media) and on artificially infected grain; thus, compactin may have good potential for practical application. The performed gene expression analysis showed compactin had a significant influence, not only on genes from DON and ZEN biosynthetic clusters, but also on the higher-order genes regulating general cell activities. As far as the authors are aware, no such studies have been carried out for other mycotoxin-inhibiting compounds. Thus, these data may be also considered to be novel. In general, the results of this study are interesting, since they demonstrate the existence of a kind of “multi-purpose” inhibitor that is able to suppress the biosynthesis of different mycotoxins without suppressing the growth of their producers. Such compounds may have some benefits for practical application because agricultural products are often contaminated with several mycotoxin-producing fungi or fungi able to produce several mycotoxins.

## Figures and Tables

**Table 1:** Effect of compactin on the zearalenone (ZEN) and deoxynivalenol (DON) production by *F. culmorum* FC-19 ^1^.

Compactin Concentration, μg/mL	Average Mycelium Dry Weight, mg	ZEN Production		DON Production	
		Average ZEN Content in the Culture Broth, μg	ZEN Production, μg/g of Dry Mycelium	Average DON Content in the Culture Broth, μg	DON Production, μg/g of Dry Mycelium
0 (Control)	661.67 ± 15.70	31.63 ± 4.01	47.74 ± 5.02	9.36 ± 0.67	14.17 ± 1.36
ZEN experiment					
25	612.67 ± 19.50	0.00	0.00	-	-
DON experiment					
10	650.00 ± 17.69	-	-	0.07 ± 0.07	0.11 ± 0.10
20	632.00 ± 11.79	-	-	0.00	0.00
50	406.01 ± 30.61	-	-	0.00	0.00

^1^ Measurements were made at the 7th day of the culture growth. The experiment was arranged in three replications; data are shown as M ± SD. LOD values for DON and ZEN were 2.0 and 0.5 ng/mL, respectively. Linear detection ranges for DON and ZEN were 5.0–400.0 and 2.0–250.0 ng/mL, respectively. The recovery for both toxins varied within 85–89%. The precision (%RSD) of HPLC measuring was 1.15 (DON) and 1.33 (ZEN).

## Data Availability

All data are shown in this manuscript and Supplementary Materials.

## Supplementary Materials

The following supporting information can be downloaded at: https://www.mdpi.com/article/10.3390/microorganisms10071347/s1, Table S1: Target genes and corresponding oligonucleotides, Table S2: Relative expression changes for genes insignificantly affected by the compactin treatment.
