# Supplementary material for: Effect of Compactin on the Mycotoxin Production and Expression of Related Biosynthetic and Regulatory Genes in Toxigenic Fusarium culmorum
Source: Microorganisms. 2022 Jul 4;10(7):1347. doi: 10.3390/microorganisms10071347 (PMC9318162; doi:10.3390/microorganisms10071347)
Supplement: Supplementary file 1 [file microorganisms-10-01347-s001.zip › microorganisms-1800599-supplementary.pdf]

Table S1. Target genes and corresponding oligonucleotides

Gene	Function	Primer sequences, 5’-3’	Probe sequences, 5’-3’	Efficiency
TRI1	Cytochrome P450 monooxygenase	F: GACATGCTGGCATATTTTGTTG<br>R: GCGGGTGGTGTGAAGGAC	BHQ1-CTCGTACCAGCGTC(FAMdT)GGGGTTGAGCAA	1.92
TRI3	15-O-acetyltransferase	F: ACAGAAGCAAACCCGTTGTTC<br>R: GACTCACAGATATGGCAAAGACTG	BHQ1-CAACAGGCGAGGACG(FAMdT)KTTGTCCGTTG	1.89
TRI4	Cytochrome P450 monooxygenase	F: ATAYGGCCCCATCGTCCG<br>R: ACGTCAAAAGCACCGACAGTG	BHQ1-GTAAACGCCCGCGAAG(FAMdT)TCACATCAGGGAT	1.96
TRI5	Trichodiene synthase	F: TGGGCACTYGTCAACG<br>R: ATCCARCATCCCTCRAAAAAG	BHQ1-<br>CCATAGTGCTACGGA(FAMdT)AAGGTTCAATGAGCAG	1.86
TRI6	Transcription factor	F: ACTATGAATCACCAACWTTCGAA<br>R: TTGTGTATCCGCCTATAGTGATC	BHQ1-CAAGGGCACCGCAC(FAMdT)GTTGGTTTGTG	1.90
TRI8	C-3 esterase	F: AACTGGGTCCTGAAAGTCTTGC<br>R: CTAGAGCAGCTCCATAGTAATTCG	BHQ1-TCCAAGTGCCTTA(FAMdT)GACTCCCCCGATGTC	2.08
TRI9	Unknown	F: AGCCGCTAAAYTGATCGACTCATA<br>R: GCTTTGGCTGCGACCCATAT	BHQ1-AGATGGACCCTGA(FAMdT)GTCTCGTGGCTCG	1.92
TRI10	Transcription factor	F: CAGCTTATCAGKTTTGAGTCTTCG<br>R: GGCCTGATTGCAAAGCTGTG	BHQ1-GGAGATTGGCGCG(FAMdT)TCACCTCCATGC	2.12
TRI11	C-15 hydroxylase	F: RCGGAAACGAGTATGATTCTCTC<br>R: GGCCWAACATGCTCTCGTTC	BHQ1-CACCCGGACGCYA(FAMdT)GAAGGATGTTCGYG	1.94

TRI12	Trichothecene efflux pump	F: AAGAGACTGCATGGATGACAGC<br>R: GGCTGCGTTCTTGTTATCTCG	BHQ1-TGGCTCAGRGCTT(FAMdT)CCTAGGAGCYATGTC	1.89
TRI14	Unknown	F: TATGGGYTATCCMGAGATGTT<br>R: RCCAGCGTGACTGAGACC	BHQ1-CCTATACAACGCCAC(FAMdT)GTCAGTACTTGGGA	1.96
TRI15	Zinc finger transcription factor	F: CCTGGCCAATGTCTCTTTTG<br>R: GCAATRAAGACATTCKCGATAATT	BHQ1-CACGGCCTATTCG(FAMdT)HCCACAGCAAGATCA	1.85
TRI101	C-3 acetyltransferase	F: GGCATTAGCGARGGAAACACA<br>R: GGCACGCCTTGGAGAGTAGAC	BHQ1-TTACAGGCTTTGGG(FAMdT)CGTYGGGACCAG	1.88
FPPS	Farnesyl pyrophosphate synthase	F: ACTGGTACAAGAAGTCTCTCGAGG<br>R: GCCTGGAGGAGCTCTGTCATC	BHQ1-CCGTCTCTCTCCTCC(FAMdT)CGAGAAGCCCC	1.93
PKS4	Polyketide synthase	F: CACATCTCCATCCAAGTTCTG<br>R: GGATCCTGCTTCAAAAAGTGT	BHQ1-GATGGTAGAAGGC(FAMdT)TGTGCATTGTACCGATC	1.91
PKS13	Polyketide synthase	F: TGGATGCGACGCCTACAC<br>R:TGCCCGTGTCGGACAATAC	BHQ1-GGCCCAACCTAC(FAMdT)CACTCGACTCGGC	1.95
PKS12	Polyketide synthase	F: AAGAGACTGCATGGATGACAGC<br>R: GGCTGCGTTCTTGTTATCTCG	BHQ1-TGGCTCACRGCTT(FAMdT)CCTAGGAGCYATGTC	1.89
AREA	Transcription factor, related to nitrogen metabolism	F: AGCATCGATGACCGTAGRACTC<br>R: ACGCCGTTTGACTGCTGG	BHQ1-GCTGATTTCTCACCACAAG(FAMdT)TCCGGCTGTC	1.97
CreA	Transcription factor, related to carbon metabolism	F: GTGACAGTTATTAAGCCCAATGG<br>R: GCCAGTGTGAGTTCGGATGTG	BHQ1-GAGTTGCCCCGACC(FAMdT)TACAAGTGCCCTCT	2.09

Pac1	pH regulatory factor	F: CTATGAGCACATCTGTGAGCGC<br>R:GGGGACGCTTGAAAGACTTG	BHQ1-GACTTGCCAA(FAMdT)GGAACTCATGCCGAACTAC	1.92
Ve1	Velvet protein	F: CAGCTTATTCGAGACTACCAAGGAG<br>R: GGGGAACTTCTTGGCGCT	BHQ1-CCTGCCGATGGCGA(FAMdT)CTTCCACCTGG	1.90
AP-1	bZIP transcription factor	F: ACAGAACGGTGGACAATTYGAC<br>R: GCTGATTGCAGTTCATGTTCTGT	BHQ1-CGCGAGCCTCAAGCCAA(FAMdT)GTTTTG	1.95
LAEA	Global regulator, a member of velvet complex	F: GCCCGAGGACGTTCCTAAC<br>R: GCGTTGGCAATCTCTTCTGAG	BHQ1-CGAGGAAGCC(FAMdT)GGCCCGCCACAGTA	1.92
MGV1	MAP kinase	F: CCAGATCCTTTGCGGTCTCA<br>R: AATCGCAAATCTTGAGCTCG	BHQ1-CTGCACCGAGATC(FAMdT)CAAGCCCGGTAACC	2.03
VELB	Velvet protein	F: ATGGGCAAGCACCGTCGT<br>R: GGGTAAGCGCGTTCTGATCC	BHQ1-AGTTCCTACGG(FAMdT)CCGCCCCAGCAATATTT	2.09
GPA1	G protein α subunit	F: ATGCTGCTGCTCGGTGCC<br>R: CATTGACTGGACGGTGTTGC	BHQ1-AAGCAGATGAAGCTTA(FAMdT)CCACGAGGGTGGC	1.91
GPA2	G protein α subunit	F: TTGCTATTAGGTTCCGGAGAGAG<br>R: TGGCCGATAGTTGAACAGCTC	BHQ1-GCGGCAAGTCGACRA(FAMdT)TGTCAAACAGATGA	2.06
GPA3	G protein α subunit	F: CGAGGACAAGGATGGGAACC<br>R: CTTGAAGAGGTCCATCTTGTTGAG	BHQ1-TGAACGAGGCGCTCA(FAMdT)GCTCTGGGAAT	2.11
GPB1	G protein β subunit	F: CCTTGCCGACACAACTCTTC<br>R: TCCTGTCGGTTGACCAGTGC	BHQ1-CGTTCGCTTGGCCTTCA(FAMdT)GAGCTGGTT	1.95

CPK1	Catalytic subunit of cAMP-dependent protein kinase A	F: GTGGATTGGTGGTCGTTGG<br><br>R: TTGCCCAATCGCTTCGTC	BHQ1-ATCCCGCATACG(FAMdT)TAACGCAGACGCCC	1.88
CPK2	Catalytic subunit of cAMP-dependent protein kinase A	F: AGTCTGGTCGCTTCCCTAACC<br><br>R: GCCATGTCGATCAAGTAGCAAG	BHQ1-TCCAAGGCTAAAG(FAMdT)GACCTCTGCCGCG	1.90
PKR	Regulatory subunit of cAMP-dependent protein kinase A	F: TAAGGTAATCAGCCAGGGAGAT<br><br>R: AGAAATTACAGTCGCAGCTCG	BHQ1-CAGCCCGGTCCCGA(FAMdT)GGTATGGGTA	1.91
ZEB1	Isoamyl alcohol oxidase	F: TGGAGAGGCCCTACCGTTG<br><br>R: GAGAACATTRTCAACACTTAAACCA	BHQ1-CGCTGGTGGCTAC(FAMdT)CTCAGGGAGGTGG	1.88
ZEB2 (ZEB2L)	Transcription factor (L isoform)	F: AAACATAGACAAAAGATGAAGCGG<br><br>R: AAGTTCACGGTATAGCAAATCATC	BHQ1-CGACGTTTGGCATTGG(FAMdT)TGTTGACTTTGGT	1.86
ZEB2 (ZEBL+S)	Transcription factor (L+S isoforms)	F: CAGCCGAAGCCATCTACGC<br><br>R: GAGGTGAGGAAGCTGTCTCTGC	BHQ1-TCCGAGAGTGGTGGTGC(FAMdT)RAAGACATCGAG	1.92
ZIF1	b-ZIP transcription factor	F: GGCCAGAATAACGTCGACTTC<br><br>R: GCAGCAACGGCAGCAGTG	BHQ1-AGTACGACGGATTCCAA(FAMdT)CTCCTGCTTCTCTC	1.85
ZRA1	ABC transporter	F: GGTTCAGATGCAGATGCCTCAC<br><br>R: TGTTCCAAGGGATCTCGGC	BHQ1-CACCCAACGATCCC(FAMdT)CTACGAAGTCCGC	1.89
RAS2	Ras-GTPase	F: CACTTTGTTGAAACTTACGACCC<br><br>R: AGCGTGACGAGATGCTGTAGAC	BHQ1-CGAATCCATTGATC(FAMdT)CGCAGTGCCGTG	2.11
FgP1	Wor1-like protein	F: GCAGCAATTAGCACACCTCC<br><br>R: CTCTCGCATCCTCGCTGAC	BHQ1-CAGCGAGAGTGGTAG(FAMdT)GCCAAGCCTGGT	1.91

Os-1	Osmosensor histidine kinase	F: ATGTTGGCCAGGAATTCAGAC<br>R: CATGGCTAGCAATTTGGCATC	BHQ1-GCAGCTTCTCTGGCT(FAMdT)GAGTGTTGCGCT	1.86
Os-2	A component of osmoregulatory MAPK pathway	F: CACATATTCACCATGGCCGAG<br>R: CCAAAAGCTCCCATGCCAAC	BHQ1-GCTGGAGATCGGAG(FAMdT)ATCTGCGCGCA	1.89
Os-4	A component of osmoregulatory MAPK pathway	F: AGACGGCGACTGAAATGAGC<br>R: CTGTAGTCCGAGGCGTGCTC	BHQ1-GCGAATGCTTCAACGA(FAMdT)TCACTCG	1.93
Os-5	A component of osmoregulatory MAPK pathway	F: CCGGAGGACTGGAAAGACC<br>R: GTAGAGTTCGGATTCATATTGCG	BHQ1-CACGCCACTCGAGTC(FAMdT)CCAAGCCATGA	1.82
Hep-1	Heterochromatin protein	F: TCCGAGCTTGTTGAAATATTTGTC<br>R: CAAAAAGCATCTCATTGACGAGG	BHQ1-GGTTGTCCTCGGGT(FAMdT)CCCAGGTCAGGT	2.08
MAP1	MAP kinase 1	F: CGACCACTGCCAGTACTTCATC<br>R: GCGCAAGACCGAAATCG	BHQ1-GTGCTGCACCGAGAC(FAMdT)TGAAGCCCTCC	1.94
FAC1	Adenylate cyclase	F: TACCAAGACAACAGTGACATTGC<br>R: GGACCTCGAGGATGATGATGTC	BHQ1-CGAGAGCGATATGC(FAMdT)GGTGACGGCTCC	1.85
FLBA	Regulator of G-protein signalling	F: GCTGATGGCTAGTGACTCGGTAC<br>R: TCACTTTCGGTTGGACCTGC	BHQ1-CCTTCGCAGCCCCAAG(FAMdT)ACGAGCAGCA	2.02
Atf1	Transcription factor	F: TCCTTGAGCGYAACCGTGTC<br>R: GCTGGGTAACAGGGCAGTCC	BHQ1-CACTGAGAACGACGC(FAMdT)CTGACGGCACAGAT	1.87
TEF1α	Translation elongation factor (reference)	F: TGGGCACTYGTCAACG<br>R: ATCCARCATCCCTCRAAAAAG	BHQ-CCATAGTGCTACGGA(FAMdT)AAGGTTCAATGAGCAG	1.90

Table S2. Relative expression changes for genes insignificantly affected by the compactin treatment

Gene	Fold change	p-value
PKS12	0.87	0.0772
Ve1	0.63	0.1207
VelB	0.91	0.2561
GPA2	1.14	0.0956
GPA3	1.22	0.2243
Os1	0.75	0.0687
Os2	0.88	0.0867
Os4	1.76	0.1776
Os5	0.93	0.9112
Ap-1	0.64	0.0733
Ras2	0.77	0.0822
Pac1	0.90	0.1587
Map1	0.69	0.0659
Hep1	0.85	0.189
LaeA	1.05	0.0787
